# Investigating the Relationship Between Dietary Antioxidant Index With Anthropometric Indices, Clinical Symptoms, Inflammatory Markers, Sleep Quality and Quality of Life in Patients With Rheumatoid Arthritis: A Cross‐Sectional Study

**DOI:** 10.1002/hsr2.72958

**Published:** 2026-08-04

**Authors:** Amirabbas Moarefian, Fatemeh Borazjani, Hadi Bazyar, Forough Nokhostin, Ahmad Zare Javid

**Affiliations:** ^1^ Student Research Committee Ahvaz Jundishapur University of Medical Sciences Ahvaz Iran; ^2^ Nutrition and Metabolic Diseases Research Center, Clinical Sciences Research Institute Ahvaz Jundishapur University of Medical Sciences Ahvaz Iran; ^3^ Department of Nutrition, School of Allied Medical Sciences Ahvaz Jundishapur University of Medical Sciences Ahvaz Iran; ^4^ Department of Internal Medicine, School of Medicine Ahvaz Jundishapur University of Medical Sciences Ahvaz Iran; ^5^ Department of Public Health Sirjan School of Medical Sciences Sirjan Iran; ^6^ Student Research Committee Sirjan School of Medical Sciences Sirjan Iran; ^7^ Faculty of Health and Life Sciences Northumbria University Newcastle upon Tyne UK

**Keywords:** cross‐sectional, Dietary Antioxidant Index, quality of life, sleep quality

## Abstract

**Background:**

Rheumatoid arthritis (RA) is a chronic autoimmune disease causing joint inflammation, pain, and deformity, significantly impacting physical and mental well‐being. Sleep disturbances and obesity exacerbate symptoms, increasing disease severity. Oxidative stress plays a key role in RA progression, and diet may influence inflammation. The Dietary Antioxidant Index (DAI) reflects dietary antioxidant intake, but its link to RA symptoms is unclear. This study explores DAI associations with RA outcomes, sleep quality, anthropometric indices, and quality of life (QoL).

**Methods:**

This cross‐sectional study included 246 RA patients attending a rheumatology clinic in Ahvaz, Iran. Dietary intake was assessed using a validated food frequency questionnaire, and DAI was calculated via the Wright method. To analyze the data, we employed various statistical methods, including analysis of variance, chi‐square tests, t‐tests, linear and Poisson regression, as well as logistic regression, as appropriate.

**Results:**

In this study, patients with RA (163 females and 83 males; mean age 50.40 ± 6.33 years) were evaluated. Poor sleep quality affected 54.9% of participants. Higher DAI scores were significantly associated with lower poor sleep quality across all models (OR = 0.66, 95% CI: 0.51–0.85). Most QoL domains showed no significant link with the DAI score, except for emotional limitations and total mental health, which improved with higher scores. Central obesity, defined by waist circumference (WC), exhibited a borderline association with DAI (OR = 0.75, 95% CI: 0.56–1.00).

**Conclusions:**

Our study suggests that among RA patients, higher adherence to DAI is linked with a lower likelihood of sleep deprivation, reduced central obesity as measured by WC, and a decreased rate of tender joints. However, no significant impact on overall QoL was observed. Further research involving larger populations is needed to confirm these findings.

**Abbreviations**
95% CI: 95% confidence interval
ALP: alkaline phosphatase
ALT: alanine aminotransferase
ANOVA: analysis of variance
AST: aspartate aminotransferase
BMI: body mass index
BUN: blood urea nitrogen
Cr: creatinine
CRP: C‐reactive protein
DAI: Dietary Antioxidant Index
DAS28: disease activity score 28
ESR: erythrocyte sedimentation rate
FFQ: food frequency questionnaire
HC: hip circumference
IPAQ: International Physical Activity Questionnaire
OR: odds ratios
RA: rheumatoid arthritis
RF: rheumatoid factor
RR: Rate ratio
SD: standard deviation
SE: standard error
SPSS: statistical package for the social sciences
VAS: visual analogue scale
WC: waist circumference
WHR: waist‐to‐hip ratio
WHtR: waist‐to‐height ratio

## 1 Background

Rheumatoid arthritis (RA) is a chronic autoimmune disease characterized by synovial joint inflammation. This leads to joint pain, swelling, erosion of cartilage and bone, and ultimately, permanent joint deformity [1, 2]. RA affects not only physical functioning but also mental well‐being and quality of life [3, 4]. Patients frequently experience chronic pain, fatigue, and limitations in daily activities, leading to issues such as depression and social isolation [5]. Approximately 60% of individuals with RA report sleep disorders [6]. These issues are often driven by persistent pain, heightened inflammatory activity, and psychological distress [7, 8, 9]. Sleep disturbances in RA are associated with worse cognitive performance, increased fatigue, and a significantly diminished quality of life [10]. Obesity exacerbates these problems, as adipose tissue acts as a reservoir for pro‐inflammatory cytokines such as TNF‐α and IL‐6 [11, 12]. Obese RA patients generally display higher disease activity scores, poorer therapeutic responses, and lower remission rates compared to those with normal body weight [13, 14, 15]. Additionally, obesity is associated with increased oxidative stress and impaired antioxidant capacity, which complicates disease management and lowers quality of life [16, 17].

Although the precise etiology of RA remains unknown, approximately half of the risk can be attributed to genetic predisposition, with environmental and lifestyle factors such as smoking, oxidative stress, obesity, and poor dietary choices also playing a role in disease onset and progression [18]. Oxidative stress, a state characterized by an imbalance between reactive oxygen species (ROS) and antioxidant capacity, is a major driver of RA pathophysiology [19]. In RA, excessive ROS production, especially in inflamed joints, leads to lipid peroxidation, cartilage degradation, and an increase in inflammation [20, 21]. This persistent oxidative damage further intensifies joint destruction and systemic inflammation, creating a harmful cycle that exacerbates both physical symptoms and emotional burden [22]. Emerging evidence highlights the role of diet in modulating oxidative stress and inflammation [23, 24, 25]. The Dietary Antioxidant Index (DAI), which takes into account the intake of vitamins A, C, and E, zinc, selenium, and magnesium, serves as a composite measure of the antioxidant potential of one's diet [26].

Research has linked diets with low DAI scores to several health conditions, including cardiovascular disease and non‐alcoholic fatty liver disease [27, 28]. On the other hand, diets high in antioxidant nutrients have been shown to reduce systemic inflammation, lower levels of pro‐inflammatory cytokines, and improve symptoms of RA and related comorbid conditions [29, 30]. Additionally, studies have indicated that individuals with higher dietary antioxidant intake report better sleep duration and quality, as well as lower levels of pain and fatigue [31]. However, to the best of our knowledge, this is the first study to examine the association between the DAI with anthropometric indices, clinical symptoms, inflammatory markers, sleep quality, and overall quality of life in individuals with RA.

## 2 Materials and Methods

### 2.1 Study Design and Participants

This cross‐sectional study was conducted on patients diagnosed with RA who were attending the outpatient rheumatology clinic in Ahvaz city, Iran, in 2024. The study was conducted in accordance with the guidelines of the Declaration of Helsinki, and the protocol was approved by the Ethics Committee of Ahvaz Jundishapur University of Medical Sciences (IR. AJUMS. REC.1403.143). Based on an estimated prevalence of insufficient sleep quality of 81% in RA patients [8], we calculated a required sample size of 237 subjects, using a 95% confidence level and a precision (d) of 5%. Accounting for an estimated 10% non‐response rate, the target sample size was set at approximately 264 subjects. All individuals were selected through simple random sampling. At the time of enrollment, participants were informed about the study's objectives, benefits, and written informed consent was obtained from each participant. RA diagnosis was established by a rheumatologist according to the 2010 ACR/EULAR classification criteria [32]. Definite RA required a total score of ≥ 6 points, confirmed synovitis in at least one joint, and no alternative diagnosis providing a better explanation. Adults aged 18–65 years with an established RA diagnosis of at least 1 year duration were eligible for inclusion. Individuals who were pregnant or lactating, those with serious comorbidities (such as heart failure, cancer, or renal failure), and patients with other connective tissue or joint diseases (including ankylosing spondylitis, lupus, and gout) were excluded. Additionally, individuals with inflammatory bowel disease, Cushing's syndrome, those who had undergone bone or joint surgery, and those adhering to specific dietary regimens were also not included. Furthermore, participants who reported a dietary intake of less than 800 or more than 4200 kcal per day (10 participants), or who did not provide adequate information regarding quality of life (3 participants) and sleep status (5 participants), were also excluded [33, 34]. In total, 246 RA patients meeting the eligibility criteria were included in this study. The current study complied with the Strengthening the Reporting of Observational Studies in Epidemiology (STROBE) checklist for cross‐sectional designs.

### 2.2 Assessment of Dietary Intakes

Dietary intake over the past year was evaluated via face‐to‐face interviews conducted by trained dieticians, employing a semi‐quantitative food frequency questionnaire (FFQ) comprising 168 items. During the interview, participants indicated how often they consumed each food, selecting from daily, weekly, monthly, or yearly frequencies. The reliability and validity of this specific FFQ had already been confirmed for the Iranian population [35]. Standard unit sizes and items reported on the household measures were converted to grams using the Household Measures Guide [36]. Energy values for the food frequency questionnaire items were derived from the USDA Food Ingredients table in the Nutritionist IV database, following modifications to account for Iranian food composition [37].

### 2.3 Assessment of Dietary Antioxidant Index (DAI)

To assess the antioxidant capacity of the diet, the DAI score was computed according to the Wright method [38]. Initially, the average global consumption levels of specific components, including vitamin A, vitamin E, vitamin C, magnesium, zinc, and selenium, were subtracted from an individual's specific intake of these components. After that, standardization of the result was achieved through division by the global standard deviation. The overall DAI score was then computed by summing the standardized values of these six components [39, 40].

### 2.4 Anthropometric Assessments

Participants' weight and height were recorded while standing in minimal clothing and no shoes. Weight was measured using a body composition analyzer (Type: GLAMOR GBF‐830, China), and height was measured with a wall‐mounted tape meter to the nearest 0.5 cm. Body mass index (BMI) was calculated by dividing weight (in kg) by height (in m) squared. Hip circumference (HC) was recorded at the widest part of the buttocks to the nearest 0.1 cm, while waist circumference (WC) was measured midway between the lower costal border and the iliac crest using a tape measure. The Waist‐to‐Hip Ratio (WHR) was calculated by dividing waist circumference by hip circumference. Similarly, the Waist‐to‐Height Ratio (WHtR) was determined by dividing waist circumference by height. Participants were classified based on general and abdominal obesity: general obesity was defined as having a BMI of 25 or higher, and abdominal obesity was assessed using several indices: a waist circumference of 88 cm or more for females and 102 cm or more for males; a waist‐to‐hip ratio of 0.8 or higher for females and 1.0 or higher for males; and a waist‐to‐height ratio of 0.5 or above for individuals under 40 years old and 0.6 or above for those 40 years old and above. Measurements below these thresholds were considered normal [41].

### 2.5 Measurement of Blood Biomarkers

After fasting for 12 h overnight, a 10 mL venous blood sample was collected from all participants. The blood samples were then centrifuged, and the resulting serum was stored at −70°C until further analysis. Levels of alanine aminotransferase (ALT), Alkaline Phosphatase (ALP), and Aspartate transaminase (AST) were determined using the method recommended by the International Federation of Clinical Chemistry [42]. All these analyses were performed using commercial kits (Pars Azmon Inc). Blood urea nitrogen (BUN) and serum creatinine (Cr) concentrations were measured using enzymatic techniques. ESR will be evaluated by the Westergren method, and Serum CRP levels and RF were measured using the quantitative kit (Pars Azmon Inc).

### 2.6 Severity of Pain

Patients' average pain levels during the previous week were measured using the Visual Analog Scale (VAS). This scale uses a 0–100 range, where higher scores correspond to more intense pain [43].

### 2.7 Disease Activity

Disease activity score 28 (DAS‐28) is a validated index for monitoring the disease activity of RA [34]. The DAS‐28 is a composite index derived from the counts of swollen and tender joints, ESR levels, and patient self‐assessment of global health via VAS. All participants underwent joint assessment by the study rheumatologist to ascertain swollen joints and tender joints counts, facilitating subsequent DAS‐28 score calculation [34, 44].

### 2.8 Quality of Life Evaluation

Quality of life (QoL) was assessed using the validated Short Form‐36 Health Status Questionnaire (SF‐36) [45]. The SF‐36 evaluates eight health‐related QoL domains: physical limitations, energy and freshness, physical performance, emotional limitations, social performance, mental health, physical pain, and general health. Two summary measures, total physical health and total mental health, are derived from distinct component scores. All dimensions score from 0 to 100, with higher scores indicating better health‐related QoL [45, 46].

### 2.9 Sleep Quality Evaluation

The Pittsburgh Sleep Quality Index (PSQI) was employed to evaluate sleep quality [47]. The index includes seven domains that capture sleep latency, quality, efficiency, disturbances, daytime dysfunction, duration, and medication use. Scores for each domain range from 0 to 3, with a total possible score of 0–21. According to previously validated cutoffs, sleep quality is considered good when the PSQI score is less than 6, and poor when the score equals or exceeds 6 [48].

### 2.10 Assessment of Other Variables

The individuals' ages, marital status, duration of disease, levels of education, job status, and smoking status were all recorded by trained interviewers. To assess physical activity, we used the globally recognized and validated Short Form International Physical Activity Questionnaire (IPAQ) [49].

### 2.11 Statistical Analysis

SPSS version 22 was employed to perform all statistical analyses. Data normality was examined using the Kolmogorov–Smirnov test prior to further statistical analysis. Participants' characteristics were compared by chi‐square or analysis of variance (ANOVA) tests across DAI quartiles for categorical and continuous variables, respectively, which were reported as a percentage or mean and standard deviation (SD). Where ANOVA indicated a significant difference, pairwise multiple comparisons were performed by means of the Least Significant Difference (LSD) post hoc test. An independent sample *t*‐test was employed to compare the mean values between the RA patient group and the global consumption values. Associations between the DAI and various continuously measured outcomes, including anthropometric indices, clinical symptoms, inflammatory markers, sleep quality, and quality of life parameters were tested by linear regression analysis and using three models, Model1: unadjusted, Model 2: adjustment for daily energy intake, Model 3: further adjustment for potential confounders including energy intake, age, sex, physical activity, job, marital status, education, body mass index, disease duration, smoking status, and medications. For outcomes representing count data, such as the number of swollen and tender joints, Poisson regression analysis was performed. Rate ratio (RR) along with 95% confidence intervals (CI) were calculated, using the first quartile of DAI as the reference. In addition, multivariable logistic regression analysis was utilized to evaluate the associations between the DAI quartiles and dichotomous outcomes including general obesity, central obesity (based on waist circumference, waist‐to‐hip ratio, and waist‐to‐height ratio), sleep quality, and rheumatoid factor (RF) status. Odds ratios (OR) with 95% CIs were calculated. *P*‐values were considered significant if they were less than 0.05.

## 3 Results

This study was conducted with 246 patients diagnosed with RA, including 83 males and 163 females, with an average age of 50.40 ± 6.33 years. It was found that 54.9% of participants had poor sleep quality. Table 1 presents the general characteristics of the study population across quartiles of DAI range score (−5.91 to 2.51). There were significant differences in marital status, education status, and duration of disease among the quartiles of DAI. Regarding inflammatory markers, CRP and ESR significantly decreased across DAI quartiles. Additionally, participants in Q4 of DAI exhibited the lowest PSQI scores compared to those in Q1. No other significant difference was observed in age, sex, job status, physical activity, smoking status, anthropometric indices, RF, VAS, DAS28, BUN, Plasma Cr, AST, ALT, ALP, and total QOL score across the DAI quartiles (*p* > 0.05).

**Table 1:** The characteristics at baseline across quartiles of DAI score in patients with rheumatoid arthritis.

Characteristics mean (SD) or (%)	DAI quartiles					*p*‐value
	Q1 (*N* = 61)	Q2 (*N* = 62)	Q3 (*N* = 62)	Q4 (*N* = 61)	Total (*N* = 246)	
DAI score	−5.91 (2.29)^a^^,^ b ^,^ c	−2.49 (0.50)^d^^,^ e	−0.88 (0.49)^f^	2.51 (2.8)	−1.69 (3.53)	< 0.001
Energy (kcal)	2055.53 (267.55)^a^	1923.00 (346.15)	1942.78 (383.41)	1979.13 (431.92)	1974.77 (363.62)	0.19
Age (years)	49.90 (6.38)	50.51 (6.09)	51.26 (5.80)	49.91 (7.03)	50.40 (6.32)	0.60
Sex (%)						0.24
Male	29.5	23.9	41.9	26.2	33.7	
Female	70.5	62.9	58.1	73.8	66.3	
Marital status (%)						0.003
Single	6.6	8.1	27.4	14.8	14.2	
Married	93.4	91.9	72.6	85.2	85.8	
Education (%)						0.04
Illiterate	8.2	22.6	27.4	18	19.1	
Elementary	9.8	16.1	19.4	19.7	16.3	
Middle‐school	21.3	22.6	25.8	27.9	24.4	
High‐school	34.4	16.1	16.1	13.1	19.9	
College	26.2	22.6	11.3	21.3	20.3	
Job (%)						0.23
Housekeeper	54.1	46.8	50	36.1	46.7	
Employee	29.5	41.9	41.9	52.5	41.5	
Retired	16.4	11.3	8.1	11.5	11.8	
P.A (met‐min/week)	868.85 (201.85)	908.39 (273.06)	857.07 (309.61)	878.00 (265.32)	878.11 (264.60)	0.73
Duration of disease (year)	2.90 (1.26)	3.35 (1.22)^e^	3.25 (1.36)^f^	2.72 (1.36)	3.06 (1.32)	0.02
Smoking (%)						0.43
Yes	8.2	11.3	17.7	11.5	12.2	
No	91.8	88.7	82.3	88.5	87.8	
Height (m)	167.41 (8.98)	164.35 (8.57)	167.03 (8.03)	164.42 (9.45)	165.80 (8.83)	0.09
Weight (kg)	75.49 (13.14)^a^^,^ c	71.29 (9.61)	71.72 (10.41)	70.35 (11.17)	72.21 (11.25)	0.06
BMI (kg/m^2^)	26.89 (3.40)	26.46 (3.61)	25.73 (3.49)	26.01 (3.46)	26.27 (3.5)	0.27
BMI categories (%)						0.37
Normal	34.4	40.3	50	41	41.5	
High	65.6	59.7	50	59	58.5	
WC (cm)	93.91 (11.24)	91.85 (11.26)	91.05 (12.33)	93.03 (11.38)	92.45 (11.55)	0.53
WC categories (%)						0.15
Normal	42.6	50	62.9	49.2	51.2	
High	57.4	50	37.1	50.8	48.8	
HC (cm)	103.24 (12.36)	103.40 (13.15)	100.83 (12.38)	102.03 (12.07)	102.37 (12.47)	0.64
WHR	0.91 (0.04)^a^	0.89 (0.04)^e^	0.90 (0.04)	0.91 (0.04)	0.90 (0.04)	0.01
WHR categories (%)						0.19
Normal	26.2	35.5	40.3	24.6	31.7	
High	73.8	64.5	59.7	75.4	68.3	
WHtR	0.55 (0.07)	0.56 (0.08)	0.55 (0.08)	0.57 (0.08)	0.56 (0.08)	0.41
WHtR categories (%)						0.85
Normal	68.9	64.5	67.7	62.3	65.9	
High	31.1	35.5	32.3	37.7	34.1	
RF (%)						0.49
Yes	78.7	82.3	71	75.4	76.8	
No	21.3	17.7	29	24.6	23.2	
VAS	38.52 (23.72)	33.38 (16.49)	37.09 (19.19)	32.62 (21.82)	35.40 (20.49)	0.31
DAS28	3.86 (0.94)	3.80 (0.7)	3.79 (0.77)	3.68 (0.85)	3.78 (0.81)	0.67
BUN (mg/dL)	15.60 (2.31)	15.61 (2.12)	15.64 (1.69)	15.36 (2.60)	15.55 (2.19)	0.88
Plasma Cr (mg/dL)	0.96 (0.14)	0.97 (0.14)	0.99 (0.13)	0.95 (0.15)	0.97 (0.14)	0.43
AST (IU/L)	27.13 (5.09)	26.74 (3.83)	26.82 (4.61)	25.90 (4.27)	26.65 (4.47)	0.47
ALT (IU/L)	36.68 (5.23)	36.37 (5.39)	35.06 (5.40)	34.85 (5.75)	35.74 (5.47)	0.15
ALP (IU/L)	97.27 (15.63)^b^^,^ c	92.79 (14.46)	90.38 (13.66)	91.57 (18.05)	92.99 (15.65)	0.08
ESR (Mm/h)	23.70 (5.46)^c^	23.66 (5.73)^e^	22.56 (5.07)	21.31 (4.47)	22.81 (5.26)	0.04
CRP (Mg/l)	15.43 (4.94)^a^^,^ b ^,^ c	13.55 (3.92)	13.56 (3.66)	13.09 (3.21)	13.91 (4.06)	0.01
PSQI score	6.44 (2.41)^b^^,^ c	5.91 (1.56)^e^	5.19 (1.74)	5.55 (1.94)	5.77 (2.07)	0.01
Sleep quality (%)						0.006
Normal	31.1	37.1	56.5	55.7	45.1	
Sleep disorder	68.9	62.9	43.5	44.3	54.9	
Total quality of life score	56.36 (13.18)	56.58 (13.27)	55.41 (13.48)	58.23 (14.05)	56.64 (13.46)	0.70
Medication						
Hydroxychloroquine(%)yes	49.2	41.9	33.9	37.7	40.7	0.35
Methotrexate(%)yes	55.7	75.8	72.6	57.4	65.4	0.03
Others(%)yes	42.6	45.1	41.9	45.9	43.9	0.61

Data are means ± SD for quantitative variables and frequency (percent) for qualitative variables.

Post hoc (LSD) according to the following pattern.

BMI categories: BMI < 25 or BMI ≥ 25. WC categories: WC ≥ 88 cm for female and WC ≥ 102 cm for male. WHR categories: WHR ≥ 0.8 for females and WHR ≥ 1 for male. WHtR categories: WHtR ≥ 0.5 for people under 40 years old and WHtR ≥ 0.6 for people over 40 years old. Sleep quality: PSQI ≥ 6 or PSQI < 6 Rf: positive or negative

Abbreviation: ALP, alkaline phosphatase; ALT, alanine aminotransferase; AST, aspartate aminotransferase; BMI, body mass index; BUN, blood urea nitrogen; Cr, creatinine; CRP, C‐reactive protein; DAI, dietary antioxidant index; DAS28, disease activity score 28; ESR, erythrocyte sedimentation rate; HC, hip circumference; PA, physical activity; PSQI, Pittsburgh sleep quality index; RF, rheumatoid factor; VAS, visual analogue scale; WC, waist circumference; WHR, waist‐to‐hip ratio; WHtR, waist‐to‐height ratio.

*From ANOVA for quantitative variables, **Chi‐square for qualitative variables.

^a^ Significant difference between the first quartile compared to the second quartile.

^b^ Significant difference between the first quartile compared to the third quartile.

^c^ Significant difference between the first quartile compared to the fourth quartile.

^d^ Significant difference between the second quartile compared to the third quartile.

^e^ Significant difference between the second quartile compared to the fourth quartile.

^f^ Significant difference between the third quartile compared to the fourth quartile.

Table 2 presents the comparison of daily energy and selected nutrient intakes in RA patients with global average daily consumption. The mean energy intake among the RA patients was significantly lower than the global average (1974.76 ± 363.61 kcal vs. 2056 ± 338 kcal, *p* < 0.001). In addition, the intake of magnesium, vitamin A, vitamin C, and zinc was also significantly lower in RA patients compared to global averages (*p* < 0.05). Conversely, selenium intake was significantly higher in the RA group than the global average (86.12 ± 37.57 µg vs. 67 ± 25.1 µg, *p* < 0.001).

**Table 2:** Comparison of daily intake of energy and components of the DAI with the global average daily consumption in rheumatoid arthritis patients.

Variable	Case group (*N* = 246)	Global consumption	*p‐value* *
Energy (kcal)	1974.76 ± 363.61	2056 ± 338	0.001
Magnesium (mg)	278.04 ± 90.45	310 ± 139.4	< 0.001
Selenium (µg)	86.12 ± 37.57	67 ± 25.1	< 0.001
Vitamin A (RE)	466.47 ± 207.37	983.9 ± 518.6	< 0.001
Vitamin C (mg)	90.84 ± 49.79	118.2 ± 43.46	< 0.001
Vitamin E (mg)	8.98 ± 4.42	8.73 ± 1.49	0.37
Zinc (mg)	8.15 ± 2.41	9.84 ± 2.19	< 0.001

Data are means ± SD for quantitative variables.

* One sample *t*‐test to compare the intake of patients with the global consumption.

Table 3 presents the associations between DAI scores and anthropometric indices, clinical symptoms, and inflammatory markers in RA patients, across three models. In model 1, higher DAI scores were significantly associated with lower body weight, BMI, WC, and HC. Regarding liver enzymes and inflammatory markers, higher DAI scores were inversely associated with AST, ALT, ALP, ESR, and CRP. Additionally, higher DAI was associated with reduced VAS scores and DAS28 scores. This association remained significant even after adjusting for potential confounders. No significant associations were observed between increased DAI score and WHR, WHtR, BUN, and creatinine levels.

**Table 3:** The association between DAI score (independent variable) with anthropometric indices, clinical symptoms, and inflammatory markers (dependent variables) in rheumatoid arthritis patients.

Variables	Model 1^a^			Model 2^b^			Model 3^c^		
	B (Unstandardized)	SE	*p‐*value	B (Unstandardized)	SE	*p‐*value	B (Unstandardized)	SE	*p‐*value*
Weight (kg)	−0.76	0.198	< 0.001	−0.76	0.198	< 0.001	−0.65	0.196	0.001
BMI (kg/m^2^)	−0.203	0.06	0.001	−0.203	0.06	0.001	−0.197	0.06	0.002
WC (cm)	−0.428	0.207	0.04	−0.428	0.208	0.04	−0.417	0.206	0.044
HC (cm)	−0.50	0.22	0.026	−0.50	0.22	0.026	−0.50	0.22	0.026
WHR	0.001	0.007	0.91	0.001	0.007	0.91	0.002	0.007	0.76
WHtR	−0.001	0.001	0.47	−0.001	0.001	0.47	−0.001	0.001	0.35
AST (IU/L)	−0.21	0.08	0.008	−0.21	0.08	0.008	−0.18	0.08	0.02
ALT (IU/L)	−0.31	0.01	0.002	−0.31	0.01	0.002	−0.31	0.1	0.002
ALP (IU/L)	−0.96	0.28	0.001	−0.96	0.28	0.001	−0.85	0.29	0.003
BUN (mg/dL)	−0.07	0.04	0.09	−0.07	0.04	0.09	−0.07	0.04	0.09
Cr (mg/dL)	−0.004	0.002	0.10	−0.004	0.002	0.10	−0.004	0.003	0.11
VAS	−0.93	0.37	0.01	−0.93	0.37	0.01	−0.67	0.31	0.03
DAS28	−0.03	0.015	0.02	−0.03	0.015	0.02	−0.02	0.013	0.05
ESR (Mm/h)	−0.36	0.09	< 0.001	−0.36	0.09	< 0.001	−0.34	0.1	< 0.001
CRP (Mg/l)	−0.25	0.07	0.001	−0.25	0.07	0.001	−0.21	0.07	0.005

Abbreviation: ALP, alkaline phosphatase; ALT, alanine aminotransferase; AST, aspartate aminotransferase; BMI, body mass index; BUN, blood urea nitrogen; Cr, creatinine; CRP, C‐reactive protein; DAI, dietary antioxidant index; DAS28, disease activity score 28; ESR, erythrocyte sedimentation rate; HC, hip circumference; PA, physical activity; RF, rheumatoid factor; VAS, visual analogue scale; WC, waist circumference; WHR, waist‐to‐hip ratio; WHtR, waist‐to‐height ratio.

* *P* < *0.05* was considered as significant.

^a^ Model 1: linear regression analysis without adjustment.

^b^ Model 2: linear regression analysis with adjustment for energy.

^c^ Model 3: linear regression analysis with correction for energy, age, sex, PA, job, marital status, education, BMI, duration of disease, smoking, and medications.

Table 4 shows the crude and multivariable‐adjusted RR and 95% CIs for swollen and tender joints across quartiles of DAI in patients with rheumatoid arthritis. For swollen joints, the crude analysis (Model 1) revealed that participants in the highest DAI quartile (Q4) had a significantly lower rate compared to the reference group (Q1), with an RR of 0.91 (95% CI: 0.73–1.12; *P*_trend_ < 0.001). Similar results were obtained in Model 2, where the RR for Q4 was 0.90 (95% CI: 0.72–1.11; *P*_trend_ < 0.001). However, after full adjustment in Model 3, the association was attenuated (RR for Q4 = 0.92, 95% CI: 0.73–1.16; *P*_trend_ = 0.32). In Model 1, the analysis for tender joints demonstrated a significant inverse association with quartiles of DAI; the RR in Q4 was 0.94 (95% CI: 0.73 to 1.21; Ptrend < 0.001). This association remained significant even after adjusting for potential confounders (RR = 0.95, 95% CI: 0.72–1.24; *P*_trend_ < 0.001).

**Table 4:** Rate ratio (95% CI) for swollen and tender joints (dependent variables) according to the quartiles of DAI (independent variable) in rheumatoid arthritis patients.

Variable	RR1 (Ref)	RR2 (CI)	RR3 (CI)	RR4 (CI)	* *p‐trend*
Swollen joints					intercept
Model 1^a^	1	0.94 (0.76–1.16)	0.97 (0.79–1.20)	0.91 (0.73–1.12)	< 0.001
Model 2^b^	1	0.92 (0.75–1.14)	0.96 (0.78–1.18)	0.90 (0.72–1.11)	< 0.001
Model 3^c^	1	0.95 (0.75–1.19)	0.93 (0.78–1.17)	0.92 (0.73–1.16)	0.32
Tender joints					
Model 1^a^	1	0.93 (0.72–1.19)	0.89 (0.69–1.15)	0.94 (0.73–1.21)	< 0.001
Model 2^b^	1	0.91 (0.71–1.18)	0.87 (0.68–1.13)	0.93 (0.72–1.20)	< 0.001
Model 3^c^	1	0.90 (0.69–1.19)	0.82 (0.62–1.10)	0.95 (0.72–1.24)	0.001

* *p* < *0.05* was considered as significant.

^a^ Model 1: Poisson regression analysis without adjustment.

^b^ Model 2: Poisson regression analysis with adjustment for energy.

^c^ Model 3: Poisson regression analysis with correction for energy, age, sex, PA, job, marital status, education, BMI, duration of disease, smoking, and medications.

Table 5 presents the associations between the DAI score, sleep quality, and QoL domains in patients with rheumatoid arthritis. In all models, higher DAI scores were significantly associated with lower PSQI scores. In Models 1 and 2, the unstandardized coefficient for PSQI was –0.15 (SE = 0.04, *p* < 0.001), and in the fully adjusted Model the coefficient was –0.13 (SE = 0.04, *p* = 0.001). For QoL subdomains, most associations were not statistically significant. However, emotional limitations exhibited a significant positive association; in Model 3, each one‐unit increase in DAI score was associated with a 1.27‐point increase in emotional limitations (SE = 0.52, *p* = 0.02). Among the composite QoL measures, total mental health was found to be significantly associated with the DAI score in the fully adjusted model (B = 0.48, SE = 0.18, *p* = 0.01), whereas the associations with total physical health (B = 0.38, SE = 0.22, *p* = 0.08) and overall QoL score (B = 0.27, SE = 0.17, *p* = 0.12) did not reach statistical significance.

**Table 5:** The association between DAI score (independent variable) with sleep quality score and quality of life parameters (dependent variables) in rheumatoid arthritis patients.

Variables	Model 1^a^			Model 2^b^			Model 3^c^		
	B (Unstandardized)	SE	*p‐*value	B (Unstandardized)	SE	*p‐*value	B (Unstandardized)	SE	*p‐*value
PSQI score	−0.15	0.04	< 0.001	−0.15	0.04	< 0.001	−0.13	0.04	0.001
Physical performance	0.28	0.39	0.48	0.28	0.39	0.48	0.31	0.30	0.29
Physical limitations	0.76	0.58	0.19	0.76	0.58	0.19	0.78	0.45	0.08
Emotional limitations	1.14	0.57	0.05	1.14	0.57	0.05	1.27	0.52	0.02
Energy and freshness	0.08	0.2	0.69	0.08	0.2	0.69	0.09	0.17	0.61
Mental health	0.25	0.15	0.10	0.25	0.15	0.10	0.20	0.16	0.22
Social performance	0.37	0.25	0.14	0.37	0.25	0.14	0.36	0.22	0.09
Physical pain	0.37	0.26	0.15	0.37	0.26	0.15	0.36	0.23	0.12
General health	0.33	0.19	0.09	0.33	0.19	0.09	0.25	0.20	0.21
Total physical health	0.39	0.29	0.18	0.39	0.29	0.18	0.38	0.22	0.08
Total mental health	0.46	0.22	0.04	0.46	0.22	0.04	0.48	0.18	0.01
Total quality of life score	0.25	0.24	0.29	0.25	0.24	0.29	0.27	0.17	0.12

*p* < *0.05* was considered as significant.

Abbreviation: BMI, body mass index; DAI, dietary antioxidant index; PA, physical activity; PSQI, Pittsburgh sleep quality index.

^a^ Model 1: linear regression analysis without adjustment.

^b^ Model 2: linear regression analysis with adjustment for energy.

^c^ Model 3: linear regression analysis with correction for energy, age, sex, PA, job, marital status, education, BMI, duration of disease, smoking, and medications.

Table 6 outlines the OR and 95% CIs for general obesity, various measures of central obesity, sleep quality, and RF status across quartiles of DAI in patients with RA. For central obesity defined by waist circumference, both Model 1 and Model 2 demonstrated no significant association with DAI quartiles; however, after full adjustment (Model 3), the association became borderline significant, with an OR of 0.75 (95% CI: 0.56–1.00, *p* = 0.05). Other anthropometric indices showed no significant associations with DAI quartiles across all models. In contrast, the associations for sleep quality were consistently significant. Participants in higher DAI quartiles had a lower odds of poor sleep quality, with the crude model showing an OR of 0.68 (95% CI: 0.54–0.86, *p* = 0.001) and similarly robust associations observed in both Model 2 and Model 3. Finally, no significant associations were observed between DAI and RF status (*p* > 0.05).

**Table 6:** Odds ratio (95% CI) for risk of general and central obesity, sleep quality, and status of RF (dependent variables) according to the quartiles of DAI (independent variable) in rheumatoid arthritis patients.

Variable	OR (CI)	B	* *p*‐value
General obesity			
Model 1^a^	0.88 (0.70–1.11)	−0.12	0.30
Model 2^b^	0.88 (0.70–1.11)	−0.12	0.29
Model 3^c^	0.87 (0.68–1.11)	−0.14	0.26
Central obesity according to WC			
Model 1^a^	0.88 (0.70–1.09)	−0.13	0.25
Model 2^b^	0.87 (0.69–1.09)	−0.14	0.22
Model 3^c^	0.75 (0.56–1.00)	−0.29	0.05
Central obesity according to WHR			
Model 1^a^	1.00 (0.79–1.27)	0.00	1.00
Model 2^b^	0.99 (0.77–1.26)	−0.01	0.92
Model 3^c^	0.30 (0.06–1.45)	−1.21	0.13
Central obesity according to WHtR			
Model 1^a^	1.07 (0.85–1.36)	0.07	0.55
Model 2^b^	1.06 (0.84–1.35)	0.06	0.60
Model 3^c^	0.93 (0.72–1.22)	−0.06	0.62
Sleep quality			
Model 1^a^	0.68 (0.54–0.86)	−0.39	0.001
Model 2^b^	0.66 (0.52–0.83)	−0.42	0.004
Model 3^c^	0.66 (0.51–0.85)	−0.42	0.001
Status of RF			
Model 1^a^	0.89 (0.68–1.16)	−0.12	0.38
Model 2^b^	0.87 (0.67–1.14)	−0.14	0.32
Model 3^c^	0.93 (0.70–1.24)	−0.07	0.61

Central obesity: WC ≥ 88 cm for female and WC ≥ 102 cm for male, WHtR ≥ 0.5 for people under 40 years old and WHtR ≥ 0.6 for people over 40 years old, and WHR ≥ 0.8 for females and WHR ≥ 1 for male. General obesity: BMI < 25 or BMI ≥ 25.

Sleep quality: PSQI ≥ 6 or PSQI < 6.

Rf: positive or negative.

Abbreviation: BMI, body mass index; DAI, dietary antioxidant index; RF, rheumatoid factor; WC, waist circumference; WHR, waist‐to‐hip ratio; WHtR, waist‐to‐height ratio.

* *p* < 0.05 statistically significant by Multivariable Logistic Regression.

^a^ Model 1: linear regression analysis without adjustment.

^b^ Model 2: linear regression analysis with adjustment for energy.

^c^ Model 3: linear regression analysis with correction for energy, age, sex, PA, job, marital status, education, BMI, duration of disease, smoking, and medications.

## 4 Discussion

To the best of our knowledge, this is the first study to comprehensively examine the relationship between DAI and anthropometric indices, clinical symptoms, inflammatory markers, sleep quality, and QoL in patients with RA. Our study found that RA patients with higher DAI scores were at significantly decreased odds of central obesity defined by WC, poor sleep quality, and risk of tender joints. Additionally, every one‐unit increase in the DAI is linked with notable reductions in weight, BMI, both waist and hip circumferences, liver enzyme levels (AST, ALT, ALP), ESR, and CRP, as well as improvements in clinical disease activity as reflected by lower VAS and DAS28 scores. However, we did not observe a significant association between DAI score and quality of life, BUN, and Cr.

Our study revealed that RA patients with higher DAI scores had significantly lower odds of central obesity, as defined by WC, although no significant association was found with general obesity. According to findings by Wang et al. [50], an inverse association existed between the Composite Dietary Antioxidant Index (CDAI) and the prevalence of general as well as abdominal obesity. This association suggests that the CDAI may function as a protective factor against inflammatory conditions caused by obesity. Additionally, Hermsdorff et al. [51] found that participants in the highest tertile of dietary total antioxidant capacity (TAC) had a WC measurement that was 4 cm smaller and exhibited lower abdominal adiposity compared to those in the lowest tertile. Furthermore, Doo et al. [52] suggested that increased consumption of dietary antioxidant vitamins may help reduce the risk of obesity. Additionally, our results demonstrated that each one‐unit increase in the DAI was linked to lower body weight, BMI, HC, and WC. These findings are consistent with the study by Aminnejad et al. [39], which showed that adolescents with higher intakes of vitamins A, C, E, zinc, and selenium had lower BMI and waist circumference. Moreover, a cohort study by Bahadoran et al. [53], conducted among healthy adults, indicated that waist circumference was significantly lower in the highest quartile of TAC compared to the lowest quartile. While many of these studies align with our findings, indicating an inverse association between dietary antioxidants and obesity, further research is needed, particularly to examine the relationship between DAI and anthropometric indices to confirm our results.

Our results suggest that RA patients who adhere to an antioxidant‐rich diet are less likely to experience poor sleep quality. This finding is consistent with the study conducted by Chen et al. [54], which found that higher CDAI scores were associated with a reduced risk of circadian rhythm sleep disorders, along with depression and short sleep duration. Additionally, cross‐sectional studies indicate that higher Dietary Antioxidant Quality Scores (DAQS) are linked to lower odds of unhealthy sleep patterns among cancer survivors [55]. Similarly, another cross‐sectional study involving Iranian postmenopausal women revealed that dietary total antioxidant capacity (DTAC) was inversely related to sleep disorders [56]. A cross‐sectional study involving 1,444 Americans found that individuals with better sleep quality reported a 37% higher daily consumption of fruit servings compared to those with poorer sleep quality [57]. Additionally, an Italian randomized controlled trial discovered that the combination of zinc, melatonin, and magnesium improved sleep quality in individuals with primary insomnia [58]. In contrast, our results, along with a few other studies, did not find a significant association between dietary antioxidants and sleep quality [52, 59]. This discrepancy may be attributed to differences in study populations and variations in the adjustment for confounding factors.

Our results indicated that a high DAI score was significantly associated with improved mental health; however, we did not observe a meaningful association with the overall QoL score among RA patients. In contrast, the findings from Ma et al. [30] suggest that increasing antioxidant intake through diet or supplements may help alleviate symptoms and enhance the quality of life in RA patients. This discrepancy may be due to differences in the assessment of dietary antioxidants and variations in the adjustment for confounding factors. Additionally, a study conducted by Wu et al. [60] on patients with heart failure indicated that dietary antioxidant insufficiency was linked to poorer quality of life. Additionally, Kou et al. [61] demonstrated that regular use of vitamin E supplements can help RA patients reduce edema, joint discomfort, and stiffness, thereby improving their QoL. Furthermore, previous studies have reported that consuming foods or dietary supplements rich in antioxidants can enhance physical activity, increase functional capacity, and improve vitality in RA patients [62].

We assessed disease activity, inflammatory markers, and joint involvement among RA patients. Our results demonstrated that increased DAI scores were significantly associated with lower VAS scores, decreased disease activity scores, lower ESR and CRP levels, and a lower risk of tender joints; these results reveal the important role of dietary antioxidants in the pathology of RA. In a clinical trial conducted by Jalili et al. [63], it was found that an antioxidant supplement containing zinc, selenium, vitamin C, vitamin A, and vitamin E significantly improved the disease activity score; however, it did not affect the number of painful or swollen joints. Additionally, other studies have shown that vitamin E supplements can help individuals with RA reduce joint discomfort and improve DAS28, ESR, and RF [61]. Furthermore, an analysis of patients living in a selenium‐rich environment in Enshi, China, indicated that those with higher selenium levels exhibited milder symptoms of RA and lower levels of inflammatory markers [64]. Moreover, vitamin C plays a key role in collagen production, a process that is crucial for preserving the health of both skin and joints [65]. In essence, vitamin C serves a dual purpose as both an antioxidant and a contributor to collagen synthesis, making it a multifaceted factor in the complex landscape of RA [66]. Overall, based on our results and these studies, antioxidants can improve the condition of RA patients [67, 68]; however, further research is needed to explore the relationship between DAI and RA.

The exact mechanisms by which antioxidants affect obesity remain unclear. However, antioxidants may have beneficial effects on weight loss. Sources of antioxidants could influence BMI by modifying lipid and carbohydrate metabolism, regulating appetite, and altering the secretion of adipocytokines [69]. Another potential mechanism by which antioxidant intake may help prevent obesity is through the regulation of brown adipose tissue metabolism and the enhancement of thermogenesis, which can help control body weight [70]. Additionally, the micronutrients and antioxidants found in fruits and vegetables play a crucial role in controlling oxidative stress and inflammation [71, 72, 73]. Furthermore, many varieties of fruits and vegetables may contain melatonin [74]. This substance can enhance sleep quality due to its natural antioxidant properties and its ability to modulate the circadian cycle [75]. The potential of dietary antioxidants to decrease rheumatoid arthritis risk operates via various biological mechanisms. Primarily, they mitigate oxidative stress by neutralizing ROS, leading to reduced inflammation. Secondly, they regulate key signaling pathways; for instance, suppressing nuclear factor kappa B (NF‐ΚB) activation diminishes inflammatory cytokine synthesis, thereby altering RA pathogenesis. Additionally, certain antioxidants, such as vitamin E, may decrease the risk of RA by enhancing gastrointestinal function [30].

Beyond its association with current disease activity and quality of life, oxidative stress has important prognostic implications in RA. Chronic oxidative imbalance contributes to endothelial dysfunction, arterial stiffness, and accelerated atherosclerosis, leading to increased risk of major adverse cardiovascular events (MACE) over time [76]. In this context, surrogate metabolic indices such as the triglyceride‐glucose (TyG) index have been successfully used to predict long‐term mortality and cardiovascular outcomes in high‐risk populations [77]. Our study introduces DAI as a similar surrogate marker of oxidative stress. Although our cross‐sectional design precludes causal inference, we propose that lower DAI may not only reflect worse current disease activity and sleep quality but also identify RA patients at higher risk for future cardiovascular complications [78].

However, several limitations should be noted. First, due to the cross‐sectional design of the study, we cannot infer causality between the DAI and outcomes such as sleep quality, anthropometric indices, and QoL. Therefore, the observed associations should be interpreted as correlational rather than causal. The possibility of reverse causality cannot be ruled out; for example, greater RA severity, functional limitations, or medication use may influence dietary choices and consequently affect the dietary antioxidant index. Moreover, potential bidirectional relationships may exist among oxidative imbalance, adiposity, sleep disturbances, and dietary patterns. Second, self‐report bias may have influenced our results because data on diet, sleep quality, and QoL were collected via questionnaires. Third, residual confounding could exist from unmeasured or unknown factors, for example, the use of dietary supplements. Additionally, since our study focused exclusively on RA patients from Ahvaz, caution is warranted when generalizing our findings to other populations. Further longitudinal and interventional research is essential to elucidate the causal impact of the DAI on clinical outcomes among RA patients.

## 5 Conclusion

In conclusion, this cross‐sectional study suggests that among RA patients, greater adherence to the DAI is associated with a reduced likelihood of sleep deprivation, central obesity as measured by WC, and tender joint rate, while no significant association was found with QoL. Further research involving larger populations is needed to confirm these findings.

## Author Contributions

**Amirabbas Moarefian:** writing – original draft. **Fatemeh Borazjani:** writing – review and editing, funding acquisition, investigation, conceptualization, methodology, validation; visualization, resources, supervision, data curation. **Hadi Bazyar:** software, formal analysis, project administration, supervision, writing – review and editing, methodology. **Forough Nokhostin:** writing – review and editing. **Ahmad Zare Javid:** writing – review and editing.

## Ethics Statement

All participants provided informed written consent. The study protocol was approved by the local Ethics Committee of Ahvaz Jundishapur University of Medical Sciences (IR. AJUMS. REC.1403.143).

## Consent

Informed consent was obtained from all participants involved in the study.

## Conflicts of Interest

The authors declare no conflicts of interest.

## Declarations

All authors have read and approved the final version of the manuscript. Dr. Fatemeh Borazjani had full access to all of the data in this study and takes complete responsibility for the integrity of the data and the accuracy of the data analysis.

## Transparency Statement

Dr. Fatemeh Borazjani affirms that this manuscript is an honest, accurate, and transparent account of the study being reported; that no important aspects of the study have been omitted; and that any discrepancies from the study as planned (and, if relevant, registered) have been explained.

## Data Availability

The Corresponding author, Fatemeh Borazjani, affirms that this manuscript is an honest, accurate, and transparent account of the study being reported; that no important aspects of the study have been omitted; and that any discrepancies from the study as planned (and, if relevant, registered) have been explained. The data that support the findings of this study are available from the corresponding author upon reasonable request. The data that support the findings of this study are available from the corresponding author upon reasonable request.
